# Sociodemographic Barriers to Physical Activity and Healthy Diet Through Social Networks in Mexican Breast Cancer Survivors

**DOI:** 10.7759/cureus.47678

**Published:** 2023-10-25

**Authors:** Sandra A. Sedano-Ochoa, María Teresa Álvarez Bañuelos, Sandra A. Saldaña-Ibarra, Omar Arroyo Helguera, Rocío Coutiño Rodríguez

**Affiliations:** 1 Public Health Institute, Universidad Veracruzana, Xalapa, MEX

**Keywords:** education, physical activity, diet, social networks, breast cancer

## Abstract

Introduction

Female breast cancer (BC) survivors are affected by poor eating habits and physical inactivity due to certain environmental, physical, and social barriers to healthy lifestyles. This study aimed to identify the sociodemographic, physical, and economic barriers hindering the adoption of physical activity (PA) and a healthy diet, as well as providing insights into how BC survivors cope with these barriers using social networks.

Methods

A cross-sectional mixed-methods study was conducted, with a self-administered questionnaire and open-ended questions to determine the barriers to PA and healthy eating, while in the second phase, an interpretive qualitative study was carried out with semi-structured interviews. Descriptive statistics, odds ratios (ORs), correspondence analysis, and multivariate analysis were used to estimate the association between moderate to vigorous PA and fruit and vegetable consumption and BC covariates.

Results

During the COVID-19 lockdown, 150 Mexican BC survivors were studied. The multivariate analysis showed that age (OR = 2.7, 95% CI: 1.0 to 7.03), socioeconomic level (OR = 3.2, 95% CI: 1.3 to 8.2), and overweight (OR = 3.6, 95% CI: 1.5 to 9.7) were significantly associated with low schooling. BC diagnosis of less than three years and age > 40 years were associated with lack of exercise. Survivors individually addressed the challenges associated with BC without the support of specialists. As a result, they sought information on social networks.

Conclusions

Regarding BC survivors, age > 40 years, low socioeconomic status, and being overweight were important gaps to PA and a healthy diet. In the testimonials, the primary obstacle to engaging in PA was lack of time, while the high cost of food was the most frequently cited reason for not following a healthy diet. Many of the individuals maintained a poor diet with a low intake of fruits and vegetables. Thus, appropriate information must be provided using technologies to develop skills to deal with BC.

## Introduction

Lifestyle is a factor that increases the risk of dying from breast cancer (BC) [1]. An inadequate diet and a sedentary lifestyle alter cell function, while a healthy diet provides protective micronutrients [1,2]. The available evidence suggests that BC survivors who closely follow a prudent dietary pattern may improve their anthropometric measures and weight, which in turn contribute to positive effects on their overall health and longevity [3]. Another aspect to consider is physical activity (PA), which may improve the immune response [4]. Lifestyle changes, including those related to diet and PA, along with socioeconomic disparities and the educational level of cancer survivors, have the potential to mitigate the long-term effects of treatment protocols. [5]. Recently, the benefits of moderate-intensity and resistance exercise during and after BC treatment have continued to be well-documented [6]. These benefits include improvements in physical condition, fatigue, emotional well-being, and the likelihood of relapses [7].

Additionally, most of the population does not meet dietary recommendations, as there is an excessive intake of added sugars and saturated fats, and a low intake of fruits and vegetables [8]. All of this has an impact on the reduction of the probability of survival [4]. Furthermore, there are factors that hinder the adoption of healthy behaviors, such as sociodemographic characteristics, economic conditions [9], and the type of treatment [10], which were exacerbated by the COVID-19 health emergency [11].

This study aimed to identify the sociodemographic, physical, and economic barriers hindering the adoption of PA and a healthy diet, as well as provide insights into how BC survivors cope with these barriers using social networks.

## Materials and methods

Design, participants, and settings

**Figure 1:**
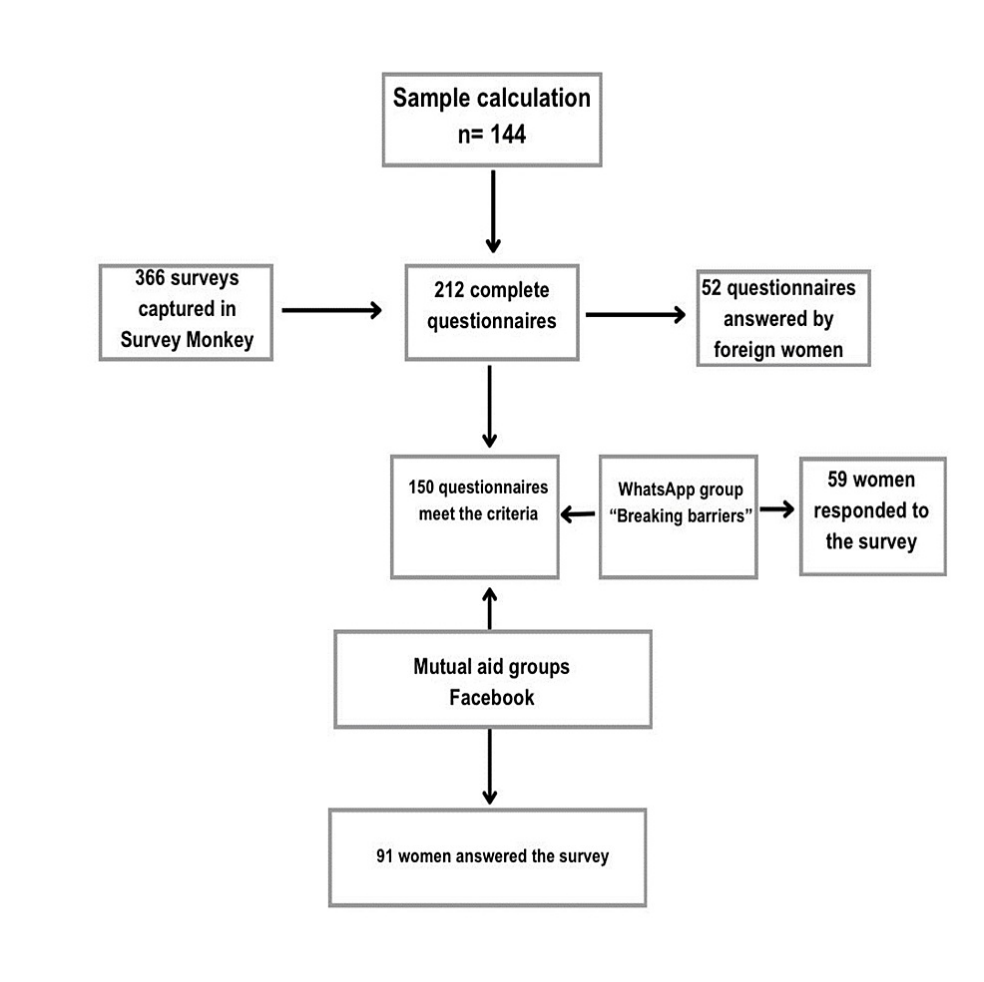
Flowchart sample

We conducted a mixed, cross-sectional, and interpretive study on female BC survivors who participated through Facebook communities and a WhatsApp group. The sample size calculation considered the main exposure variable of high school education, with a proportion of patients having a high school education or less at 39%, a confidence level of 90%, and a statistical power of 80%. Epidat 4.1 software was used for these calculations. The estimated minimum sample size was that of 144 BC survivors [12]. After the baseline was established, female participants were interviewed. Female Mexican BC survivors with access to a cell phone or computer with internet to reach mutual help groups on Facebook and WhatsApp were selected using the criteria flowchart sample selection (Figure 1).

Data collection

Data were collected using a self-administered online survey on SurveyMonkey (SurveyMonkey Inc., San Mateo, CA), which included sections on sociodemographic, economic, and PA information sourced from the following validated questionnaires: the WHO STEPwise Approach to Noncommunicable Diseases Risk Factor Surveillance (STEPS) [13] and the Frequency of Food Consumption of Adolescents and Adults survey, elaborated by the National Health and Nutrition Survey 2016 (ENSANUT, 2016) [14]. Overweight and obesity were defined using the body mass index (BMI) classification proposed by the WHO [15]. Two open-ended questions about barriers to PA and a healthy diet were asked. Considering the testimonies from the female participants, we explored how they faced the barriers and obstacles to engaging in PA and maintaining a healthy diet.

Variables

PA was measured using the relationship between the working metabolic rate per hour and the resting metabolic rate (METs) of 1.0 kcal x kg x hour. A sedentary lifestyle was defined as 1-1.5 METs (greater than six hours sitting), light activity as 1.6-2.9 METs, moderate as 3-5.9 METs, and vigorous as ≥6 METs [16]. A healthy diet was defined as consisting of 2 ½ to three cups of vegetables and 1 ½ to two cups of fruit, and the type of fat consumed to determine food portions. Standardized images with equivalent measurement units were included in the questionnaire [8].

Demographic variables included age (<40 years and ≥40 years), basic schooling level (primary and secondary) and maximum (high school or higher), employment status (worker or non-worker), and place of residence defined as rural (<2,500 inhabitants) or urban (≥2,500 inhabitants) according to the classification of the National Institute of Statistics and Geography [17], alcohol consumption (defined as one standard drink, approximately 14 g of alcohol/day), and smoking (defined as one cigarette a day or more and categorized as current, former, or non-smoker).

Statistical analysis

A descriptive analysis was carried out for the sociodemographic variables, which were represented by frequencies and percentages. In the univariate analysis, the odds ratio (OR) and the 95% confidence intervals (CI) were calculated. A simple correspondence analysis (two variables) and multiple correspondence analysis (more than two variables) were carried out, allowing for a graphic table to show the relationship between the variables: adequate consumption of fruits and vegetables, low socioeconomic level, marital status, PA, and diagnosis time. In the multivariate analysis, the dependent variable was a low level of schooling, while the other covariates were independent. The analysis was performed using the statistical package IBM SPSS version 25.0 (IBM Corp., Armonk, NY). A word cloud was created to analyze open-ended questions with RStudio software (RStudio Team, Boston, MA).

The central category for qualitative data was defined as coping, which was made up of beliefs, the tendency to action, behavior, an incentive to make changes, and attitudes toward the pandemic. Spectral analysis of speech was performed and the rating was described in terms of consistency, variability, and extreme case to identify the differences and similarities between the survivors on how they coped with the gaps during the COVID-19 lockdown. Thus, MAXQDA software version 20.0.7 (VERBI GmbH, Berlin, Germany) was used for this analysis.

Ethics

The Statement of Ethics study was approved by the ethics committee of the Instituto de Salud Publica, Universidad Veracruzana, under registration number CEI-ISP-R14/2020. Female participants agreed to be involved in the research prior to the study with written informed consent.

## Results

Quantitative results

Participants Characteristics

A total of 150 female participants from 23 states were recruited, with a greater number representing the central part of the country. The average age was 45.9 years with 8.6 ±SD. with a minimum of 28 and a maximum of 65 years. The main characteristics of female participants studied include 97.3% (n = 146) residing in urban areas. Female participants with a high level of schooling and socioeconomic status predominated, and a similar proportion stated to have a job. Overweight and obese female participants represented 42.0% (n = 63).

Statistically Significant Results

When comparing the survivors with the level of schooling (low and maximum) with the sociodemographic and anthropometric characteristics, the following results were significant: socioeconomic level (p = 0.001), time of diagnosis, and reusing frying oil (Table 1).

**Table 1:** Comparison of some general characteristics, lifestyle, diet, and time of diagnosis with respect to the level of education in breast cancer survivors BMI: body mass index. Low: <18.5 kg/m2; normal: 18.5 to 24.9 kg/m2; overweight: 25 to 29.9 kg/m2; obesity: >30 kg/m2. MET: metabolic equivalents; METs per hour. Sedentary: 1-1.5 METs (> six hours sitting); light activity: 1.6-2.9 METs; moderate: 3-5.9 METs; vigorous: ≥6 METs. Proportions were compared by the chi-square test as %.

	Basic		Superior		
Variables	n = 29 (%)		n = 121 (%)		p
Age					
<40 years	11	(37.9)	29	(24.0)	0.046
>40 years	18	(62.1)	92	(76.0)	
Place of residence					
Rural	3	(10.3)	1	(0.8)	0.004
Urban	26	(89.7)	120	(99.2)	
Time of diagnosis					
<3 years	28	(96.6)	97	(80.2)	0.03
>3 years	1	(3.4)	24	(19.8)	
Socioeconomic status					
Low level	17	(58.6)	33	(27.3)	0.001
High level	12	(41.4)	88	(72.7)	
Current BMI classification					
Low	1	(3.4)	1	(0.8)	0.005
Normal	8	(27.6)	77	(63.6)	
Overweight	18	(62.0)	38	(31.4)	
Obesity	2	(7.0)	5	(4.2)	
Physical activity (METs)					
Sedentary	17	(58.6)	59	(48.8)	0.62
Light	2	(7.0)	19	(15.7)	
Moderate	5	(17.2)	23	(19.0)	
Vigorous	5	(17.2)	20	(16.5)	
Sedentary lifestyle					
<6 hours	18	(62.0)	75	(62.0)	0.99
>6 hours	11	(38.0)	46	(38.0)	
Vegetable consumption					
Adequate	6	(20.7)	20	(16.5)	0.59
Inadequate	23	(79.3)	101	(83.5)	
Fruit consumption					
Adequate	8	(20.7)	43	(36.0)	0.42
Not suitable	21	(79.3)	78	(64.0)	
Cooking fat					
Oil	27	(93.1)	120	(99.2)	0.04
Butter	2	(6.9)	1	(0.8)	

A low level of schooling was associated with low socioeconomic status (p = 0.015, OR = 3.2), overweight prior to diagnosis (p = 0.005, OR = 3.6), and in borderline values, age over 40 years (Table 2).

**Table 2:** Risk factors associated with low level of schooling in women breast cancer survivors in the multivariate analysis Multivariate logistic regression analysis, model adjusted for the following variables: fruit consumption, housework activity, and time of diagnosis by the “forward: conditional” method. OR: odds ratio; 95% CI: confidence interval at 95%. Overweight: > 25 kg/m2. Dependent variable: level of schooling (level of basic education = primary and secondary).

Variable	Unadjusted		Adjusted	
	OR (CI 95%)	p	OR (CI 95%)	p
Age > to 40 years	2.7 (1.08-5.4)	0.034	2.7 (0.99-7.1)	0.051
Rural area	0.07 (0.07-0.72)	0.07	0.11 (0.09-1.3)	0.08
Low socioeconomic status	3.7 (1.63-8.7)	0.01	3.2 (1.3-8.2)	0.015
Previous overweight	2.4 (1.10-5.7)	0.007	3.6 (1.5-9.7)	0.005
Current overweight	0.6 (0.29-1.39)	0.62	1.7 (0.53-5.3)	0.51
Tobacco use	1.02 (0.11-9.5)	0.98	1.8 (0.15-21.5)	0.64

**Figure 2:**
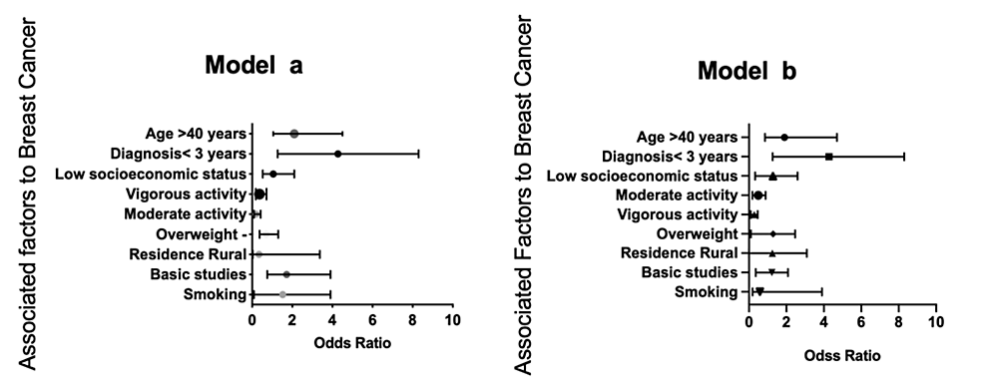
Multivariate analysis. Physical inactivity associated with sociodemographic variables, overweight, and smoking Multivariate logistic regression analysis. Model a: Intro and Model b: Forward conditional, adjusted for sociodemographic variables. OR: odds ratio; 95% CI: confidence interval at 95%. One metabolic equivalent (MET): consumption of 1 kcal/kg/h; sedentary lifestyle: 1-1.5 METs (> six hours sitting); light activity: 1.6-2.9 METs; moderate: 3-5.9 METs; and vigorous: ≥6 METs.

Other factors associated with BC and PA were related, while age > 40 years and diagnosis less than three years were significantly associated as risk factors (Figure 2). In the adjusted model, only the diagnosis greater than three years was maintained (OR = 0.3 (0.1-0.7)).

**Figure 3:**
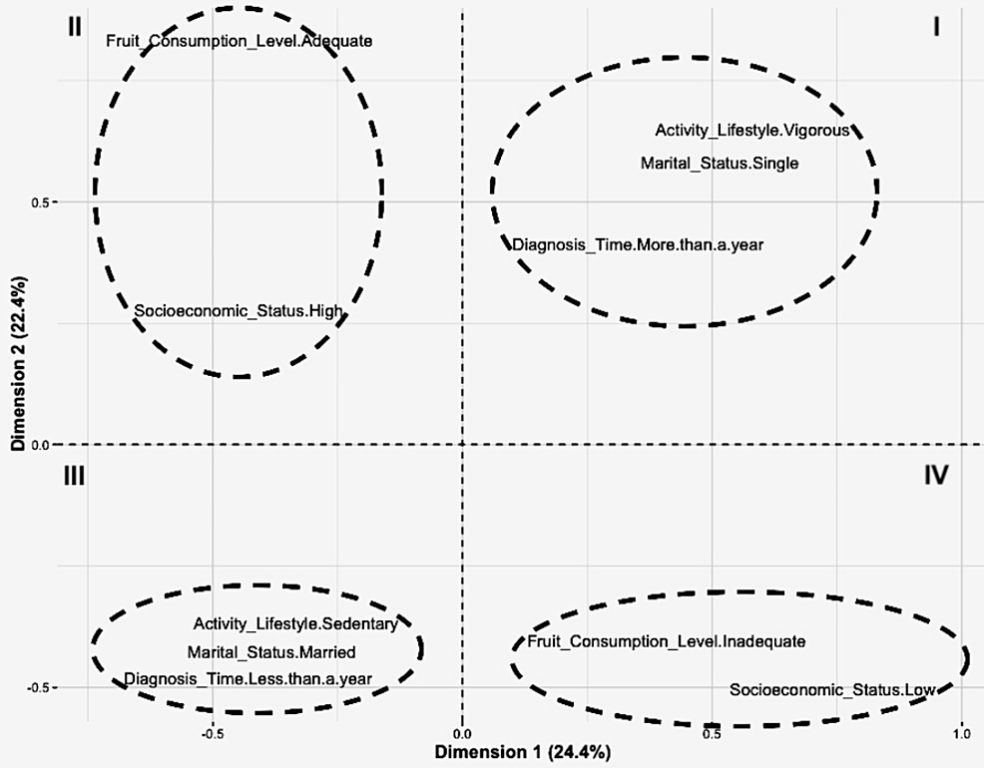
Analysis of simple and multiple correspondence of the behavior of the variables with respect to their categories Correspondence analysis between simple (I and IV) and multiple (II and III). Variable DX: Breast Cancer Diagnosis. One metabolic equivalent (MET): consumption of 1 kcal/kg/h; sedentary lifestyle: 1-1.5 METs (> six hours sitting); light activity: 1.6-2.9 METs; moderate: 3-5.9 METs; and vigorous: ≥6 METs.

In the correspondence analysis between simple and multiple variables, they were independent, although graphically in quadrants I and IV. The behavior observed between the variables regarding their categories showed a grouping predisposition between the survivors according to the consumption of fruits and their socioeconomic level. When forming three groups with PA, marital status, and diagnosis time as covariates, quadrants II and III showed correspondence with the female survivors who did vigorous PA (75 minutes) were single and had a BC diagnosis for more than one year. In quadrant IV, the relationship between a sedentary lifestyle and being married variables had correspondence with the diagnosis time of less than one year (Figure 3).

Qualitative analysis

Open-Ended Question Analysis

The most important barrier that female survivors mentioned inhibiting PA was the lack of time, fatigue, and the pandemic. Regarding the consumption of fruits and vegetables, they pointed out high costs, dissatisfaction, and lack of habit.

In-Depth Question Analysis

**Figure 4:**
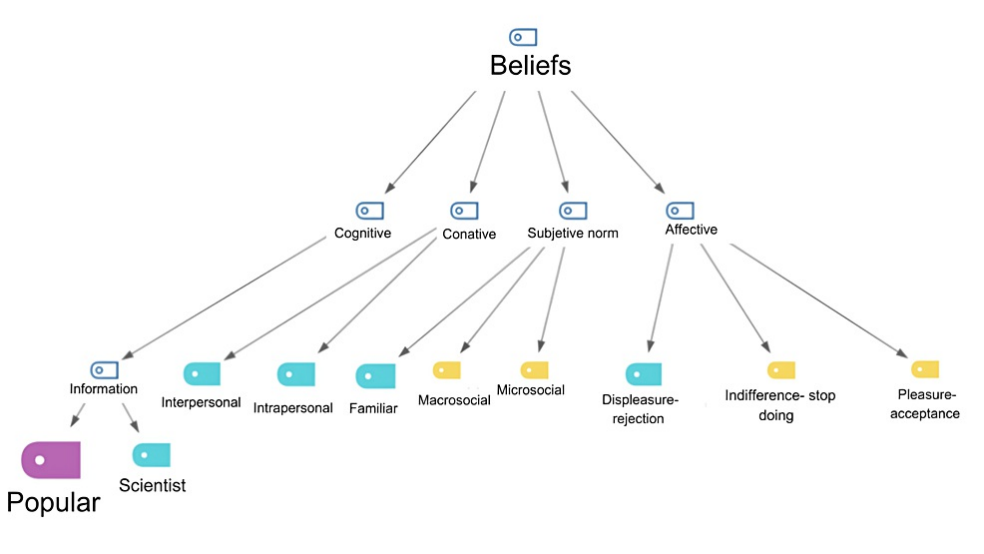
Hierarchical code-subcode model of belief Source: Created by the authors in MAXQDA software version 20.0.7.

Survivors defined their coping beliefs according to popular social media and networking sources. They mentioned that they coped with the barriers mainly using their beliefs or information gathered from popular sources (Figure 4). An example of comments collected from social groups includes: "I learned information through a Facebook group because my oncologist never told me about diets or anything else" (Clavel). The statement relates to group rehabilitation since they received support in mutual-help groups. The advice given by colleagues in the WhatsApp group was: “At least go for a walk, don't lie down all the time"(Azucena). Alternatively, they searched social networks for information: "I learned through YouTube, when I found out I had cancer I looked for information there because everything I know is because of the internet. My physician has not given me much information about it, for example, my oncologist did not tell me that I cannot exert force with the arm that was operated on. He didn't even talk to me about lymphedema, I believe it is called" (Girasol). The pandemic was a major concern, and it significantly reduced the chances of leading a healthy diet and physical exercise: "As I said, houses are very small spaces, so you cannot really go for a formal walk to do exercise. From what I've been researching when it comes to walking, you have to walk ten thousand to fifteen thousand steps, so that it can be considered one hour of exercise at a constant speed. It's very difficult to get these hours of walking at home, isn’t it?” (Lotus).​​

## Discussion

Despite the proven benefits of a nutrient-rich diet to prevent cancer, our study showed that during the lockdown, participants emphasized that the cost and distaste for vegetables and fruits were important gaps to eating them, which is consistent with Cho et al. [18], who reported that a greater attraction to foods high in saturated fats and the high cost of healthy foods were important barriers.

The economic, schooling, and health insurance conditions of women in Mexico are a determining influence on social inequalities in the options available to control and treat BC. In this study, the low economic level meant an important barrier to the proper consumption of fruits and vegetables. The results of this research yield evidence supporting that individuals with low economic levels purchased low-cost food to satisfy their caloric needs instead of fruits and vegetables regardless of schooling level [19]. Among Mexican households that exhibited negative changes in their diet, 66% reduced their intake of meat and fish, while over 50% reported a decreased intake of fruits and vegetables [20]. In addition, socioeconomic levels were significantly associated with the low schooling of survivors and represented an important risk factor. Thus, the negative influence of low economic income on the survival of this disease is verified and reiterated.

Our results showed an important association between basic education, being older than 40 years of age with socioeconomic level, and BMI prior to diagnosis <25 kg/m2. It is worth mentioning that the level of schooling is an important element for healthy behavior, and although in this study almost all the survivors had a basic education level, and approximately half of them had a university degree, they had low consumption of fruits and vegetables. Even though this has been proven otherwise, it would be worthwhile to explore why a high level of schooling does not guarantee to lead a healthier lifestyle.

It is important to note that the ENSANUT-COVID-19 study reported that the Mexican population is characterized by body weight exceeding the recommended weight, including a high prevalence of overweight and obese women, mainly in Mexico City and in states located in the center part of the country, which is not in line with the data yielded by the cohort of women studied [11]. However, it is consistent with what has been reported by the literature worldwide that weight loss favors overall survival [21]. Similarly, a low level of schooling was related to the lack of physical activity (p = 0.005), which led to becoming overweight before developing the disease.

Likewise, the results suggest that the recent diagnosis of BC is associated with both a high prevalence of a sedentary lifestyle and a significant relationship between a diagnosis time of fewer than three years, and being married also contributed to a sedentary lifestyle.

It is unavoidable not to mention that these results were influenced by the COVID-19 lockdown, since that recreational/leisure physical activities were reduced during the pandemic. Also, outdoor spaces were limited, such as gyms and recreational equipment, among other restrictions to prevent the spread of the virus [22]. The current paradigm shift due to the pandemic caused by the SARS-CoV-2 virus has mainly affected vulnerable groups like this one. As evaluated in a different study [23], the pandemic increased the levels of insecurity and anxiety in patients with BC and also has affected the preventive and curative decision-making process for patients.

From a qualitative perspective, our study continues to reflect on the barriers and unattended needs of BC survivors. Moreover, it provides an overview of the physical and economic obstacles that affect the healthy practices of survivors during the current pandemic, since we found that women lack the skills to exercise due to the sequelae of treatments, fatigue, depression, and family difficulties, which is line with narratives of exploratory analysis as the physical, psychological, and social impact experienced by women with this disease was revealed [7]. To deal with the lockdown, social networks, particularly Facebook groups, represented the first line of emotional support for vulnerable groups [24], as they interacted with other BC survivors and shared knowledge and information for a greater and better chance of remaining cancer-free. Our study notes the lack of comprehensive support, which was compensated with peer support and provided them with greater security to carry out their treatments. Self-help groups have a positive impact on women struggling with BC, as they increase their knowledge, skills, and confidence needed to handle challenges. Furthermore, those groups can facilitate the development of new self-awareness and promote acceptance [25].

Our study confirms that survivors faced these problems individually with invalid information that they obtained mainly from internet pages. However, an intervention study showed that a high percentage of women said that the media is an important information source.

It is highlighted that health professionals provide no comprehensive support for survivors to promote self-care skills, and in this case, it was partially compensated by the support of other women living the same situation, which provided them with greater security to go through their treatments, and the painful conditions of treatments were mitigated. This is in line with a group intervention that was effective in mitigating the feeling of loneliness in women with BC after six months of diagnosis. It is important to stress that family and professional support, in addition to a correct comprehensive orientation through nutrition and exercise, plays a key role in overcoming the difficulties that survivors have to cope with [26].

There were some limitations. Survivor involvement depended on the stage they were at after their diagnosis. Some were in the grieving process, so the openness of the female participants was limited when collecting their testimonies.

There is the possibility of a trend in the sample collection since we had access to a group of women with a high socioeconomic status who had cell phones and computers with internet and not those from undeveloped areas, where it is more difficult to have access to these electronic devices.

## Conclusions

This research is one of the few mixed-methods studies that obtained a representative sample of the Mexican population, so it was possible to further explore the problem. It was conducted in the unique context of the current pandemic, which represents a total paradigm shift in the face of both cancer survival and the benefits of using technology and social networks, which also allows a larger scope. It is recommended to implement public policies to promote a comprehensive approach to treating survivors considering the various conditions experienced by patients.

Age over 40 years, low socioeconomic status, and being overweight were confirmed to be important gaps to healthy behaviors among BC survivors. Additionally, lack of time, recent diagnoses, and the pandemic were important limitations to exercise.
